# Forced Remote Learning During the COVID-19 Pandemic in Germany: A Mixed-Methods Study on Students' Positive and Negative Expectations

**DOI:** 10.3389/fpsyg.2021.642616

**Published:** 2021-08-31

**Authors:** Thomas Hoss, Amancay Ancina, Kai Kaspar

**Affiliations:** Department of Psychology, University of Cologne, Cologne, Germany

**Keywords:** COVID-19, higher education, student perception, ease-of-retrieval effect, mixed-methods study, remote learning

## Abstract

The COVID-19 pandemic poses great challenges to higher education. Universities had to change their infrastructure to full remote teaching and learning environments in a very short time. Lecturers and students were forced to adjust their established routines and concepts of teaching and learning. During the first nationwide lockdown in Germany, we explored students' anticipations regarding the risks and chances of this challenging situation. They were asked about the negative and positive effects of this sudden switch to online university courses and the relevance personally ascribed to each of these expected effects. A sample of 584 students provided 3,839 statements, which were examined by means of qualitative content analysis. While 57.7% of the statements concerned negative effects, 42.3% dealt with positive ones. The range of expected negative and positive effects was wide, but key themes emerged particularly frequently. While the mentioned effects were generally considered to be of high personal relevance, negative effects were rated as significantly more relevant, but with only a small effect size. The relevance of negative effects was considered higher by master students than by bachelor students. Relevance ratings were significantly higher for the first effect mentioned compared with all subsequent effects, indicating an ease-of-retrieval effect, which is relevant from both a methodological and content perspective. The results provide important insights into students' perspectives on remote learning that will be significant beyond the current pandemic, as they can guide sustainable measures by exploiting opportunities and mitigating risks. We discuss practical implications and methodological limitations of the study.

## Introduction

Since early 2020, the COVID-19 pandemic has had a considerable influence on society (Nicola et al., 2020). The first nationwide lockdown in Germany in March 2020 forced universities to change their infrastructure to fully remote teaching and learning environments in a short period of time. Lecturers and students were forced to adjust their established routines and concepts of teaching and learning (Shapiro et al., 2020). The lack of appropriate infrastructure, skills, and experience with remote teaching and learning had already been noted previously (Persike and Friedrich, 2016). While media use in Germany increased rapidly from 2012 to 2015, the use of digital learning media stagnated (Dolch and Zawacki-Richter, 2018). Especially in higher education, students' media use does not necessarily correlate with digital learning, given that digital media are rarely an integral part of teaching and learning (Persike and Friedrich, 2016). Despite the fact that university administrations ascribe high relevance to digitalization processes, they were quite reserved when it came to assessing the status quo of digitalization in 2018 (Gilch et al., 2019). Indeed, the transition that universities usually “must undergo to adapt to online program delivery involves many complex issues” (Amirault, 2012, p. 253). Digital and remote learning measures are complex projects consisting of numerous conceptual and procedural steps that require substantial planning and preparation time (Rüth and Kaspar, 2017). Past research identified key factors that determine remote learning readiness in higher education, such as technological infrastructure, policy makers, financial aspects, human resources, but most importantly the skills and attitudes of teachers and learners (Rohayani, 2015). A survey at a large German university showed that the majority of student teachers does not perceive any learning opportunities for the acquisition of corresponding competences (Jäger-Biela et al., 2020).

Against the background of this unpreparedness regarding remote teaching and learning and due to the unpredictable duration and course of the pandemic, students may develop pessimistic expectations regarding their forthcoming study activities. Indeed, research shows that the pandemic has led to anxiety, distress, and uncertainty among German adults in general (Benke et al., 2020; Petzold et al., 2020). International studies revealed that the pandemic has had several negative impacts on students' well-being and psychosocial variables (Cao et al., 2020; Wang and Zhao, 2020), and that it has led to job losses or reduced income (Aucejo et al., 2020) as well as to new obligations and challenges in family life (Ayuso et al., 2020). However, remote teaching and learning can also have positive effects for students, for example, increased flexibility regarding the time and place of learning (Arkorful and Abaidoo, 2015). Also, students could expect positive impacts on the quality of learning materials and some long-term benefits to the universities' digital infrastructure (Getto and Kerres, 2017). It can be assumed that the transition to remote teaching and learning offers both opportunities and challenges (Adedoyin and Soykan, 2020). Students' expectations regarding remote learning have already been examined (e.g., Mupinga et al., 2006), but previous research focused on specific online courses and referred to existing infrastructure. In contrast, the present study focused on unprepared and forced remote learning during the COVID-19 pandemic, a situation in which students' specific expectations were mostly unknown. Hence, during the first nationwide lockdown in Germany in 2020 we asked:

**RQ1**: What negative and positive effects on their own study activities do students expect, given the abrupt transition to remote teaching and learning?

Irrespective of the quality of effects, the relevance personally ascribed to the expected effects may vary. People tend to give more weight to negative over positive information (Kanouse, 1984). Especially young people show pronounced negativity biases at the attention and memory level (Kaspar et al., 2015). Moreover, people tend to overestimate recent (negative) experiences (Kuchler and Zafar, 2019). Indeed, the pandemic situation is especially characterized by negative effects on individuals. Petzold et al. (2020) found that half of over 6,000 German respondents suffered from anxiety or psychological distress in connection with the COVID-19 pandemic. According to another study, anxiety and depression in young German adults were directly related to the reduction of social contacts and perceived changes in everyday life (Benke et al., 2020). Consequently, we assumed that negative effects would be mentioned more often and perceived as more personally relevant than positive effects:

**H1**: The number of expected negative effects is higher than the number of expected positive effects.
**H2**: Negative effects are perceived as more personally relevant than positive effects.

From a methodological perspective, when examining students' expectations, the order in which effects are mentioned is noteworthy: The fact that a certain negative/positive effect comes to mind first may suggest that it is more relevant than other effects subsequently mentioned. Indeed, the ease-of-retrieval hypothesis assumes that people use the ease with which information comes to mind as a heuristic applied to subsequent judgments (Schwarz et al., 1991; Menon and Raghubir, 2003). Similarly, Wänke and Hansen (2015) hold that the relative experience of fluency in cognitive processing is an important diagnostic cue for judgment formation. Therefore, we hypothesized that the perceived relevance of negative/positive effects decreases across effects mentioned by the students:

**H3**: There is a decrease in the perceived personal relevance of expected effects across responses.

## Materials and Methods

### Participants

This study is the first part of a larger survey conducted in April and May 2020. The median duration to complete this survey part was 393.50 s. Incentives to participate were not provided. Conditions of participation were a minimum age of 18 years and enrollment at a German university. Students enrolled at distance-learning universities were excluded. The final sample contained 584 students from different universities (*M*_*age*_ = 24.07 years, *SD*_*age*_ = 4.88; 496 women, 82 men, 6 diverse); 403 students were in a bachelor's degree program and 181 were in a master's degree program. The sample was heterogeneous regarding the study program, but most of the participants (404) were enrolled in a teacher training program covering a range of scientific disciplines, 71 were studying psychology, and 38 were enrolled in a media-related program.

### Measures

The students provided informed consent and sociodemographic variables (age, gender, study program, university). Subsequently, they were asked to state up to five negative and positive effects they expect from the transition to remote teaching and learning (maximum 150 characters per statement). The instructions were as follows: “The coronavirus pandemic necessitates an (almost) complete conversion of university courses to a remote mode. Please indicate up to five negative/positive effects that you currently expect for your own studies due to this changeover.” Given that the sudden outbreak of the pandemic, with all the resulting constraints and repercussions on everyday life and study activities, primed a rather negative mindset, we first asked all participants to name the expected negative effects. Afterwards, we explicitly asked them to reflect on potential positive effects. The students additionally rated the effects mentioned in terms of relevance, using a scale ranging from 1 (hardly relevant) to 5 (highly relevant).

### Coding and Analysis

We performed a qualitative content analysis based on the standard approach by Mayring (2015), following previous works (Kaspar et al., 2010, 2014): Firstly, the transcribed responses were paraphrased. Next, a category system was inductively and iteratively developed by deriving the categories from the first 10% of the material covering 3,839 short statements in total. This resulted in two systems, one for negative and one for positive effects. Subsequently, the inter-coder reliability was evaluated to ensure the applicability and objectivity of the category systems. For this purpose, two persons independently coded the same material after prior introduction to both category systems. Inter-coder reliability was initially calculated after coding 10% of the material to detect possible sources of errors and to optimize the category systems accordingly. Once the category system had been optimized, the entire material was coded and the inter-coder reliability was quantified by Kappa (Cohen, 1960), indicating very good agreement with a minimum κ = 0.88 across all categories and mentioned effects. In rare cases of disagreement, a consensual agreement was subsequently achieved through discussion in order to allow frequency analyses.

## Results

### Expected Effects (RQ1)

Table 1 (negative effects) and Table 2 (positive effects) present the kind of effects expected by the students and associated quantities.

**Table 1:** Category descriptions, number of statements in descending order, and example statements for negative effects.

**Category of negative effects**	***n***	**Example statements**
Decreased quality of teaching and learning, including performance assessment	304 (246)	“impairment of comparing performances in courses,” “monotone tasks instead of interesting discussions on a topic,” “practice-based courses like project seminars could lose quality”
General decrease or lack of social interaction and communication	300 (242)	“communication will be difficult,” “negative effects on networking,” “social life in the university ceases”
Fear of less contact, interaction with, and support from other students	208 (200)	“limited exchange with fellow students,” “I cannot meet my fellow students and the start of the semester is socially impeded,” “difficult small group work and cooperation with fellow students”
Negative effects on course of studies, including study time extension	192 (150)	“study is taking longer as not all lectures will be offered online,” “cancellation of practical courses,” “postponement of exams”
Closure of university facilities and services and hence impeded access to university resources	180 (153)	“problems accessing literature due to the libraries being closed,” “no public spaces for self-studying,” “exercise rooms are closed,” “no possibility to borrow media equipment”
Problems in self-regulation in terms of self-studying, self-organization, self-discipline, and problems with structuring everyday life	168 (140)	“more individual responsibilities,” “missing structure due to the lack of presence teaching,” “more organization is required,” “more independent work (self-study with texts)”
Perceived uncertainty due to a lack of information	141 (126)	“uncertainty how it will go on,” “no detailed information on the part of the university and institutes,” “poor communication of the university”
Increased effort required for studying in terms of time and workload	129 (114)	“higher performance requirement to replace presence,” “increased time-based effort,” “significantly more effort due to unfamiliar situation”
Less contact and interaction with lecturers and university staff and reduced feedback and support	117 (110)	“no possibilities for personal contact with the lecturer,” “no personal supervision of the bachelor thesis,” “fewer opportunities for questions to the lecturer,” “reduced addressability of lecturers”
Decreased motivation in study activities and learning	69 (67)	“lacking motivation for digital learning,” “decrease in motivation,” “it is harder to get up”
Lack of an appropriate working environment	62 (59)	“no home office is possible due to noise,” “inadequate working environment at home,” “no suitable room for video conferencing”
Technological problems on the student side, including hardware and software	57 (53)	“unstable internet connection at home,” “no well working laptop,” “technology and internet is not sufficiently available to everyone”
General problems and concerns regarding technology	48 (47)	“technical problems,” “technical challenges,” “technical services and malfunctions”
Negative impacts of working environment on health	26 (25)	“headache and eye pain, because of being on the computer the whole day,” “I miss activity, due to the omission of the ways to university,” “too little physical activity in everyday life”
Lack of clear separation between study activities and private life	21 (21)	“increased mixing of private life and work, less relaxation,” “worse separation between study and leisure time,” “parallel childcare”
Financial problems	19 (17)	“financial losses,” “I lost 1700€ due to a canceled field trip,” “financial problems due to elimination of the part-time job”
General negative affect	10 (9)	“more stress of missing out,” “feeling nervous,” “adapting to unfamiliar situation is characterized by fear”
Technological problems regarding the stability of digital services	9 (9)	“congested online systems, for instance Zoom,” “accessing problems to learning platforms,” “LMS is overloaded”
Other unspecific or rare statements not fitting any of the categories above	155 (134)	“less concentration on the essential,” “no face-to-face lectures”

*Column n shows the number of statements and in brackets the number of students who provided at least one statement of the respective category (i.e., corrected for multiple responses of individual students falling into the same category)*.

**Table 2:** Category descriptions, number of statements in descending order, and example statements for positive effects.

**Category of positive effects**	***n***	**Example statements**
Saving of time	325 (290)	“long way to university is spared,” “more free time due to the omission of commuting,” “saves time because everything can be done from home”
Increased flexibility in time and work management	306 (268)	“flexible division of time,” “greater time-related flexibility participating in lectures,” “free division of the workload”
Gain in media- and study-related competences and skills	217 (169)	“knowledge acquisition about digital media, also for the time after the pandemic,” “getting to know methods and applications for digital teaching, for me as a trainee teacher”
Hope for progress of digitalization in universities and society	142 (124)	“university finally gets up to the newest technological standard,” “advance in digitalization,” “further development of technology and associated possibilities,” “it is timely to digitize education”
Increased flexibility in the (asynchronous) reception and processing of course materials	107 (99)	“lectures and seminars are still available afterwards,” “you can stop videos of lectures and listen to important parts multiple times,” “multiple viewing of lectures”
Hope for better work-life balance due to remote learning	103 (95)	“easier combination of study, work, and household,” “facilitates support of persons in need of care in the household,” “easier childcare”
Increased quality of digital teaching and learning	63 (58)	“useful and good materials could be created, which can be worked from home,” “digital contents are well prepared,” “more intensive examination of the learning material”
Increased flexibility in general	61 (59)	“more flexibility,” “own schedule,” “more flexibility in the course of study”
Increased flexibility in learning location	53 (52)	“participation possible when sick,” “not linked to a specific place,” “flexible place of study”
Benefits for communication and interaction	22 (22)	“written and therefore reliable communication,” “communication with lecturers until today,” “cohesion despite distance,” “more cooperation with lecturers and other students,”
Financial gains and savings during the challenging pandemic situation	20 (19)	“saving of money,” “no costs for driving,” “can go to work more often,” “save money by spending less on entertainment or other recreational activities”
Staying healthy	18 (18)	“no risk of infection,” “social distancing,” “prevention of corona being spread,” “no internal conflicts regarding infection risks”
Slowing down and more sleep	18 (18)	“deceleration,” “less stress,” “no obligation to get up early,” “sleep longer”
Protection of the environment	7 (7)	“protection of environment due to omission of traveling,” “less use of paper,” “better for the environment”
Increased motivation in study activities and learning	5 (5)	“more motivation participating in lectures, due to no commuting,” “learning motivation due to videos”
Other unspecific or rare statements not fitting any of the categories above	157 (128)	“strong connection to studies,” “you wear more comfortable clothes”

*Column n shows the number of statements and in brackets the number of students who provided at least one statement of the respective category (i.e., corrected for multiple responses of individual students falling into the same category)*.

Among the negative effects associated with the transition to remote teaching and learning, the most frequently mentioned effects concerned social interaction and communication: The students provided 300 statements indicating a general decrease or lack of social interaction and communication. A fear of less contact, interaction with, and support from other students was expressed in 208 further statements, whereas 117 additional statements predicted less contact and interaction with lecturers and university staff as well as reduced feedback and support. It is to be noted that many statements addressed psychosocial variables, namely problems with self-regulation in terms of self-studying, self-organization, self-discipline, and problems with structuring everyday life (168), perceived uncertainty due to a lack of information (141), increased effort required for studying in terms of time and workload (129), decreased motivation in study activities and learning (69), and a lack of clear separation between study activities and private life, including childcare (21). A further substantial category are negative expectations regarding the quality of remote teaching and learning, including performance assessment (304), followed by negative effects on the course of studies, including study time extension (192), and the closure of university facilities and services and hence impeded access to university resources (180). Moreover, the students mentioned general problems and concerns regarding technology (48), technological problems on the student side, including hardware and software (57), but only rare problems regarding the stability of digital services (9). Several statements addressed the lack of an appropriate working environment (62) and negative effects on health (26). A general negative affect was stated 10 times. Finally, some statements (19) referred to financial problems.

Regarding positive effects of remote teaching and learning, almost one third of all statements referred to increased flexibility, including flexibility in time and work management (306), in the (asynchronous) reception and processing of course materials (107), in learning location (53), and an increased flexibility in general (61). The largest single category was the saving of time (325), but many statements also expressed a gain in media- and study-related competences and skills (217), hope for progress in digitalization in universities and society (142), hope for a better work-life balance due to remote learning (103), and slowing down as well as getting more sleep (18). Only some statements reflected an expected increase in the quality of digital teaching and learning (63), financial gains and savings during the challenging pandemic situation (20), increased motivation in study activities and learning (5), and protection of the environment (7). A few statements indicated benefits for communication and interaction (22). Importantly, only 18 statements addressed the underlying reason for the transition to remote teaching and learning, namely staying healthy.

### Number of Negative and Positive Effects (H1)

Overall, 2,215 negative effects were mentioned, which corresponds to 57.7% of all statements, in contrast to 1,624 positive effects (42.3%). The students reported between zero and five negative and positive effects, respectively. On average, each student stated 3.79 (*SD* = 1.40) negative effects and 2.78 (*SD* = 1.56) positive effects. A *t*-test for paired samples showed that this difference was significant, *t*_(583)_ = 14.08, *p* < 0.001, *d* = 0.58. Hence, the ratio of negative to positive effects was unequal in favor of the negative effects. There were no significant differences between bachelor and master students regarding the number of reported negative effects, *t*_(582)_ = −1.19, *p* = 0.236, *d* = −0.11, and positive effects, *t*_(582)_ = 1.23, *p* = 0.220, *d* = 0.11. More details on the categories most frequently mentioned by the two student groups are depicted in Table 3.

**Table 3:** Comparison of bachelor and master students' five most mentioned categories for negative and positive effects.

	**Most mentioned negative effects**		**Most mentioned positive effects**	
	**Bachelor**	**Master**	**Bachelor**	**Master**
1.	General decrease or lack of social interaction and communication (13.7)	Decreased quality of teaching and learning, including performance assessment (15.5)	Saving of time (20.9)	Saving of time (17.8)
2.	Decreased quality of teaching and learning, including performance assessment (12.9)	General decrease or lack of social interaction and communication (13.2)	Increased flexibility in time and work management (19.3)	Increased flexibility in time and work management (17.8)
3.	Fear of less contact, interaction with, and support from other students (9.4)	Closure of university facilities and services and hence impeded access to university resources (11.3)	Gain in media- and study-related competences and skills (12.4)	Gain in media- and study-related competences and skills (15.6)
4.	Negative effects on course of studies, including study time extension (9.3)	Fear of less contact, interaction with, and support from other students (9.4)	Hope for progress of digitalization in universities and society (8.1)	Hope for progress of digitalization in universities and society (10.2)
5.	Problems with self-regulation in terms of self-studying, self-organization, self-discipline, and problems with structuring everyday life (8.8)	Negative effects on course of studies, including study time extension (7.2)	Increased flexibility in the (asynchronous) reception and processing of course materials (7.2)	Hope for better work-life balance due to remote learning (7.2)

*Numbers in brackets indicate how many percent of the total number of negative/positive statements fall into the respective categories*.

### Personal Relevance of Expected Effects (H2, H3)

We analyzed the relevance that students personally ascribed to the effects mentioned. Based on 527 students who provided at least one negative and one positive effect accompanied by relevance ratings, we compared the average relevance of negative effects (*M* = 3.97, *SD* = 0.69) and positive effects (*M* = 3.77, *SD* = 0.91), *t*_(526)_ = 3.95, *p* < 0.001, *d* = 0.17. Supporting H2, negative effects were perceived as more personally relevant, but with a small effect size. Relevance of negative effects was considered higher by master than by bachelor students, *t*_(402.89)_ = 2.78, *p* = 0.006, *d* = 0.24. No significant difference between student groups was found regarding positive effects, *t*_(537)_ = −0.32, *p* = 0.751, *d* = −0.03.

To test H3, postulating a decrease in the perceived personal relevance across the effects mentioned, ANOVAs for repeated measures (Greenhouse-Geisser) were separately computed for positive and negative effects. A prior MANOVA, including relevance ratings of five negative and five positive effects, indicated a significant result (*p* < 0.001), but it was based on a greatly reduced sample size as only a few students reported ten effects in total. When inserting five levels to the ANOVAs (for five effects rated), we found a significant result for negative effects, *F*_(3.90, 1, 033.42)_ = 15.42, *p* < 0.001, $\eta_{\text{p}}^{2}$ = 0.06. As expected, the first effect mentioned was considered to be more relevant than any of the other four subsequent effects, all *p*s < 0.001 (Bonferroni-adjusted). No further significant differences were found. Similarly, relevance ratings of positive effects also decreased, *F*_(3.71, 451.96)_ = 6.81, *p* < 0.001, $\eta_{\text{p}}^{2}$ = 0.05; in this case the relevance of the first effect was higher than the relevance of the third, fourth, and fifth effect each, all *p*s ≤ 0.005. When only the first three effects were included, the number of students in the ANOVAs substantially increased (Table 4) and replicated the aforementioned results, except that the relevance of the second positive effect was also significantly lower compared to the first effect. These relative results were complemented by *t*-tests comparing the observed mean values against the scale's midpoint. Mean personal relevance was above the scale's midpoint for all positive and negative effects mentioned. Effect sizes were medium to large, but higher for negative effects (Table 4).

**Table 4:** Students' ratings of the relevance personally ascribed to negative and positive effects expected from the abrupt transition to remote teaching and learning.

**Order of effects**	**Relevance of negative effects**						**Relevance of positive effects**					
	**Descriptive statistics**			**One-sample** ***t*** **-test**			**Descriptive statistics**			**One-sample** ***t*** **-test**		
	***M***	**SD**	***n***	***t***	***P***	***d***	***M***	**SD**	***n***	***t***	***p***	***d***
1.	4.27	0.94	560	32.21	<0.001	1.36	3.98	1.09	538	20.81	<0.001	0.90
2.	3.97	1.06	545	21.28	<0.001	0.91	3.89	1.06	451	17.95	<0.001	0.85
3.	3.97	1.00	478	21.25	<0.001	0.97	3.79	1.16	314	12.08	<0.001	0.68
4.	3.85	1.06	357	15.22	<0.001	0.81	3.73	1.21	185	8.22	<0.001	0.60
5.	3.84	1.16	270	11.91	<0.001	0.73	3.60	1.25	124	5.27	<0.001	0.47

*Personal relevance of the stated effects was measured on a scale ranging from 1 (hardly relevant) to 5 (highly relevant). One-sample t-tests were computed against the scale's midpoint of 3. Sample size n decreases across the order of effects, as students were asked to name up to five effects per valence*.

Given that the first effect mentioned by the students was rated as personally most relevant, which effects were stated first? Regarding negative effects, students most frequently mentioned a general decrease or lack of social interaction and communication (90), followed by negative effects on the course of study (77), and a decrease in the quality of teaching and learning (60). Regarding positive effects, students most often referred to time-saving aspects (172), followed by more flexibility in time and work management (132), and a gain in media- and study-related competences (64). A complete overview is presented in the Supplementary Material.

## Discussion

### Central Findings and Implications

In spring 2020, the COVID-19 pandemic forced teachers and learners to abandon familiar teaching and learning routines, and universities were suddenly required to create remote study environments. The perspective of students on this new role of remote learning has also been examined quantitatively by Adnan and Anwar (2020), however, with a limited scope on specific topics. A qualitative approach was taken by Irawan et al. (2020) via unstructured phone interviews, but this study covered only a small sample. The present study examined students' expectations by means of a mixed-methods but structured approach and based on a large sample. We gathered 3,839 statements from German students reflecting negative and positive effects related to forced remote learning and the relevance personally ascribed to these effects. Our key findings are as follows:

Firstly, students expected a wide range of effects, but the negative effects outnumbered the positive ones. This result may reflect the overall negative impact of the pandemic, which was already perceived at the time of the study in spring 2020 (Petzold et al., 2020). There was heightened anxiety among students at this time (Wang and Zhao, 2020). This result is also in line with known forms of negativity bias in the domains of attention and memory (Kaspar et al., 2015) and information weighting (Kanouse, 1984; Kuchler and Zafar, 2019).

It is striking that many expected negative effects expressed a decrease or lack of social interaction and communication with others, including fellow students and lecturers. Correspondingly, Adnan and Anwar (2020) found that students ascribe high relevance to face-to-face contact even in remote learning settings. Past research indicates that communication, interactivity, and social aspects are considered as quality criteria of digital learning (Masoumi and Lindström, 2012). Indeed, counseling and support by lecturers in remote learning settings were found to be predictors of course satisfaction and learning achievement (Paechter et al., 2010). Hence, social isolation is a critical problem and must be adequately addressed in remote learning. Another substantial number of statements reflected anticipated problems with self-regulation in terms of self-studying, self-organization, self-discipline, and problems with structuring everyday life. Indeed, self-regulation is crucial for performance in remote leaning settings in general (Sharma et al., 2007) and it can be supported by characteristics of the learning environment such as usefulness and interactivity (Liaw and Huang, 2013). However, in times of a pandemic, many areas of everyday life are severely restricted and usual routines do not take hold, so that increased demands are placed on the ability to self-regulate. Also, many statements expressed perceived uncertainty due to a lack of information. Mupinga et al. (2006) already found that obtaining course information in advance is of great importance in remote learning. However, the abrupt change from offline to remote learning during the pandemic was unpredictable and this need was not met. As a consequence, many students also expected negative effects on the quality of remote teaching and learning, including performance assessment. It therefore seems imperative to verify whether the measures taken at universities have been able to dispel these concerns in the meantime. The present study thus represents a valuable reference for upcoming success evaluations.

The most frequently mentioned positive effects expected from the transition to remote learning were the saving of time as well as flexibility issues regarding time and work management, the (asynchronous) reception and processing of course materials, choice of learning locations, and in general. These advantages of remote learning had already been highlighted previously (Daymont et al., 2011), but during the current pandemic, these benefits seem particularly important for adequately addressing and compensating challenges in other areas of life, such as those associated with job loss (Aucejo et al., 2020) and new obligations in family life (Ayuso et al., 2020). Hence, universities and lecturers should try to maximize flexibility options during this challenging time. Interestingly, many students also expressed the hope that they will experience an increase in their media- and study-related competences and skills. Indeed, universities where face-to face teaching dominates often lack learning opportunities to acquire relevant competences and skills (Jäger-Biela et al., 2020). However, the participants' hope might be in vain, as the development of appropriate learning opportunities takes time and cannot be realized solely through a quick expansion of digital infrastructure. Further research is needed to evaluate which of the potential benefits of remote learning can be established for higher education in the long term. Many students also expressed hope for progress in digitalization in universities and society and for a better work-life balance due to remote learning. Hence, they identified current weaknesses in the digital infrastructure in Germany (Gilch et al., 2019) and emphasized the long-term potentials of remote learning for their work-life balance. Interestingly, only few statements addressed the protective effects of remote learning regarding SARS-CoV-2, the virus that causes COVID-19. Perhaps this aspect is so self-evident that students did not find it worth mentioning.

When comparing the positive and negative effects, some overlaps are recognizable: While some statements reflect negative expectations regarding the quality of teaching and learning, other statements reflect quite the opposite. University measures must therefore be designed to realize opportunities and mitigate risks in terms of quality. This requires a precise evaluation of the conditions for success and failure in remote teaching and learning, in order to continuously improve ongoing measures. In this context, it seems to be a fruitful strategy to include the students' perspective in the design of digital teaching and learning in the sense of a co-creational process (Lubicz-Nawrocka, 2018). Also, some aspects mentioned by the students are counteractive: While flexibility ranked highly among the positive effects, having to face self-regulation issues were prominent among the anticipated negative effects. These aspects, however, are inextricably linked. Self-regulation is positively related to students' achievements in remote learning and their learning experience (Artino, 2008; Paechter et al., 2010). Hence, universities should implement specific measures to foster the different determinants of self-regulated learning (Boekaerts, 1999) so as to unfold the positive effect of the flexibility provided by remote teaching and learning.

Importantly, the effects mentioned by the students were—on average—personally considered to be highly relevant. Negative effects were rated significantly more relevant than positive effects, although this difference was characterized by a small effect size. Also, master students considered the negative effects as more relevant than bachelor students. This could be explained by the fact that master students already have more established study routines and the pandemic-related change management process might seem more challenging for them from this perspective.

Finally, and as expected, the relevance ratings decreased across the sequence of effects mentioned, corresponding to an ease-of-retrieval effect (Schwarz et al., 1991; Menon and Raghubir, 2003; Wänke and Hansen, 2015). Thus, the particularly important aspects are those that came to the participants' minds first. These aspects should be given special consideration in university measures, and researchers who collect qualitative data via surveys and interviews should be aware of this phenomenon in general.

### Limitations

The strength of the present study is based on the collection of immediate student responses to the first nationwide Lockdown in Germany in 2020. Important implications for current and future measures can be derived from the data obtained in this way, which would be difficult to reproduce via a retrospective survey. At the same time, some limitations are associated with this approach:

Methodologically, the rigid sequence of the effects asked about (first negative, then positive) could be seen as a limiting factor. We do not know whether the ratio of anticipated effects would change in favor of positive effects if the participants had been asked about them first or if the sequence had been randomized. The fact that the onset of the pandemic and the unprepared switch to remote learning were accompanied by an overall negative mindset, as documented by many studies from this period (Benke et al., 2020; Cao et al., 2020; Petzold et al., 2020; Wang and Zhao, 2020), argued against both of the latter design options. In general, selecting an entry topic is an inherent challenge of qualitative surveys. Therefore, we deliberately asked about negative effects first to better reflect the prevailing situation regarding the pandemic and the abrupt shift to remote learning at German universities.

Another possible limitation is that our study only addressed pre-course expectations of students. These will not necessarily have been confirmed by subsequent experiences. A continuous development of students' expectations over time and a post-course evaluation were not part of this study. At the time this study was planned and conducted, there was no indication of how long the impacts of the pandemic would last. Remote learning was still in its infancy at many German universities and a clear process was not foreseeable. Students still are confronted with an almost complete transition to remote learning today. It might be too early for a final summative evaluation of these measures, as higher education in Germany is still undergoing a digital transformation process that has been intensified by the pandemic.

Lastly, the results of this study on German students cannot be generalized to the teaching and learning conditions around the globe. Aristovnik et al. (2020) reported that the frequency of students' personal worries during the COVID-19 pandemic varies considerably across continents. Moreover, there might be substantial differences in the rating of specific effects from one university to another, and some factors may strongly depend on the individual lecturers and learners. Based on the present results, future research should take a more nuanced perspective to examine the fit between the skills and needs of individual students on the one hand, and remote teaching and learning interventions during pandemic conditions on the other.

## Data Availability Statement

The raw data supporting the conclusions of this article will be made available by the authors, without undue reservation.

## Ethics Statement

In accordance with local legislation and institutional requirements, an ethics board approval was not required for this study on human participants. In Germany, as stated by the German Research Association (DFG, https://www.dfg.de/foerderung/faq/geistes_sozialwissenschaften/index.html), the present survey study did not require the approval of an ethics committee, because the research did not pose any threats or risks to the respondents, it was not associated with high physical or emotional stress, and the respondents were informed about the objectives of the survey in advance. At the beginning of the study, participants were informed that the data of this study will be used for research purposes only and that all data are collected anonymously. Thus, no identifying information was collected. Participants who prematurely abandoned the survey were not included in the analyses and all of their data were deleted from the dataset. Informed consent to participate in this study was provided by clicking a corresponding box, and participation was voluntary in all cases.

## Author Contributions

TH, AA, and KK designed the study, performed the analyses, interpreted the results, and wrote the manuscript. TH and AA collected the data. KK organized and supervised data collection and acquired funding. All authors contributed to the article and approved the submitted version.

## Conflict of Interest

The authors declare that the research was conducted in the absence of any commercial or financial relationships that could be construed as a potential conflict of interest.

## Publisher's Note

All claims expressed in this article are solely those of the authors and do not necessarily represent those of their affiliated organizations, or those of the publisher, the editors and the reviewers. Any product that may be evaluated in this article, or claim that may be made by its manufacturer, is not guaranteed or endorsed by the publisher.

## References

[B1] Adedoyin O. B. Soykan E. (2020). Covid-19 pandemic and online learning: the challenges and opportunities. Interact. Learn. Environ. 10.1080/10494820.2020.1813180. [Epub ahead of print].

[B2] Adnan M. Anwar K. (2020). Online learning amid the Covid-19 pandemic: students' perspectives. J. Pedagog. Sociol. Psychol. 2, 45–51. 10.33902/JPSP.202026130933083098

[B3] Amirault R. J. (2012). Distance learning in the 21st century university: key issues for leaders and faculty. Q. Rev. Dist. Educ. 13, 253–365.

[B4] Aristovnik A. Keržič D. Ravšelj D. Tomaževič N. Umek L. (2020). Impacts of the COVID-19 pandemic on life of higher education students: a global perspective. Sustainability 12:8438. 10.3390/su12208438 PMC8634691 34869802

[B5] Arkorful V. Abaidoo N. (2015). The role of e-learning, advantages and disadvantages of its adoption in higher education. Int. J. Instruct. Technol. Dist. Learn. 12, 29–42.

[B6] Artino A. (2008). Promoting academic motivation and self-regulation: practical guidelines for online instructors. Tech. Trends 52, 37–45. 10.1007/s11528-008-0153-x

[B7] Aucejo E. M. French J. Araya M. P. U. Zafar B. (2020). The impact of COVID-19 on student experiences and expectations: evidence from a survey. J. Public Econ. 191:104271. 10.1016/j.jpubeco.2020.104271 32873994 PMC7451187

[B8] Ayuso L. Requena F. Jiménez-Rodriguez O. Khamis N. (2020). The effects of COVID-19 confinement on the spanish family: adaptation or change?. J. Comp. Fam. Stud. 51, 274–287. 10.3138/jcfs.51.3-4.004

[B9] Benke C. Autenrieth L. K. Asselmann E. Pané-Farré C. A. (2020). Lockdown, quarantine measures, and social distancing: associations with depression, anxiety and distress at the beginning of the COVID-19 pandemic among adults from Germany. Psychiatry Res. 293:113462. 10.1016/j.psychres.2020.113462 32987222 PMC7500345

[B10] Boekaerts M. (1999). Self-regulated learning: where we are today. Int. J. Educ. Res. 31, 445–457. 10.1016/S0883-0355(99)00014-2

[B11] Cao W. Fang Z. Hou G. Han M. Xu X. Dong J.. (2020). The psychological impact of the COVID-19 epidemic on college students in China. Psychiatry Res. 287:112934. 10.1016/j.psychres.2020.112934 32229390 PMC7102633

[B12] Cohen J. (1960). A coefficient of agreement for nominal scales. Educ. Psychol. Meas. 20, 37–46. 10.1177/001316446002000104

[B13] Daymont T. Blau G. Campbell D. (2011). Deciding between traditional and online formats: exploring the role of learning advantages, flexibility, and compensatory adaptation. J. Behav. Appl. Manag. 12, 156–175.

[B14] Dolch C. Zawacki-Richter O. (2018). Are students getting used to learning technology? Changing media usage patterns of traditional and non-traditional students in higher education. Res. Learn. Technol. 26. 10.25304/rlt.v26.2038

[B15] Getto B. Kerres M. (2017). Protagonists of digitalization in higher education: modernization or profiling?. Zeitschr. Hochschulentwicklung 12, 123–142. 10.3217/zfhe-12-01/07

[B16] Gilch H. Beise A. S. Krempkow R. Müller M. Stratmann F. Wannemacher K. (2019). Digitalization of Universities: Results of a Focus Study for the Expert Comission of Research and Innovation. (No. 14-2019). Berlin: Expertenkommission Forschung und Innovation (EFI).

[B17] Irawan A. W. Dwisona D. Lestari M. (2020). Psychological impacts of students on online learning during the pandemic COVID-19. KONSELI J. Bimbingan Konseling 7, 53–60. 10.24042/kons.v7i1.6389

[B18] Jäger-Biela D. J. Kaspar K. König J. (2020). Learning opportunities for the acquisition of digitalization-related media competences: analyses of the study offer and the usage behavior of student teachers at the University of Cologne, in Education, School, Digitalization, eds K. Kaspar, M. Becker-Mrotzek, S. Hofhues, J. König, and D. Schmeinck (Münster: Waxmann), S. 64–70.

[B19] Kanouse D. E. (1984). Explaining negativity biases in evaluations and choice behavior: theory and research. Adv. Consum. Res. 11, 703–708.

[B20] Kaspar K. Gameiro R. R. König P. (2015). Feeling good, searching the bad: positive priming increases attention and memory for negative stimuli on webpages. Comput. Human Behav. 53, 332–343. 10.1016/j.chb.2015.07.020

[B21] Kaspar K. Hamborg K.-C. Sackmann T. Hesselmann J. (2010). The effectiveness of formative evaluation in the development of usable software: a case study. Zeitschr. Arbeits Organisationspsychol. 54, 29–38. 10.1026/0932-4089/a000003

[B22] Kaspar K. König S. Schwandt J. König P. (2014). The experience of new sensorimotor contingencies by sensory augmentation. Conscious. Cogn. 28, 47–63. 10.1016/j.concog.2014.06.006 25038534 PMC4154453

[B23] Kuchler T. Zafar B. (2019). Personal experiences and expectations about aggregate outcomes. J. Finance 74, 2491–2542. 10.1111/jofi.12819

[B24] Liaw S. S. Huang H. M. (2013). Perceived satisfaction, perceived usefulness and interactive learning environments as predictors to self-regulation in e-learning environments. Comput. Educ. 60, 14–24. 10.1016/j.compedu.2012.07.015

[B25] Lubicz-Nawrocka T. M. (2018). Students as partners in learning and teaching: the benefits of co-creation of the curriculum. Int. J. Stud. Part. 2, 47–63. 10.15173/ijsap.v2i1.3207

[B26] Masoumi D. Lindström B. (2012). Quality in e-learning: a framework for promoting and assuring quality in virtual institutions. J. Comp. Assist. Learn. 28, 27–41. 10.1111/j.1365-2729.2011.00440.x

[B27] Mayring P. (2015). Qualitative Content Analysis. Basics and Techniques. Weinheim: Beltz.

[B28] Menon G. Raghubir P. (2003). Ease-of-retrieval as an automatic input in judgments: a mere-accessibility framework? J. Consum. Res. 30, 230–243. 10.1086/376804

[B29] Mupinga D. M. Nora R. T. Yaw D. C. (2006). The learning styles, expectations, and needs of online students. Coll. Teach. 54, 185–189. 10.3200/CTCH.54.1.185-189

[B30] Nicola M. Alsafi Z. Sohrabi C. Kerwan A. Al-Jabir A. Iosifidis C.. (2020). The socio-economic implications of the coronavirus and COVID-19 pandemic: a review. Int. J. Surg. 75, 185–193. 10.1016/j.ijsu.2020.04.018 32305533 PMC7162753

[B31] Paechter M. Maier B. Macher D. (2010). Students' expectations of, and experiences in e-learning: their relation to learning achievements and course satisfaction. Comp. Educ. 54, 222–229. 10.1016/j.compedu.2009.08.005

[B32] Persike M. Friedrich J.-D. (2016). Learning With Digital Media From a Student Perspective. Arbeitspapier Nr. 17. Berlin: Hochschulforum Digitalisierung.

[B33] Petzold M. B. Bendau A. Plag J. Pyrkosch L. Mascarell Maricic L. Betzler F.. (2020). Risk, resilience, psychological distress, and anxiety at the beginning of the COVID-19 pandemic in Germany. Brain Behav. 10:e01745. 10.1002/brb3.1745 32633464 PMC7361063

[B34] Rohayani A. H. (2015). A literature review: readiness factors to measuring e-learning readiness in higher education. Proc. Comput. Sci. 59, 230–234. 10.1016/j.procs.2015.07.564

[B35] Rüth M. Kaspar K. (2017). The e-learning setting circle: first steps toward theory development in e-learning research. Electron. J. e-Learn. 15, 92–101.

[B36] Schwarz N. Bless H. Strack F. Klumpp G. Rittenauer-Schatka H. Simons A. (1991). Ease of retrieval as information: another look at the availability heuristic. J. Pers. Soc. Psychol. 61, 195–202. 10.1037/0022-3514.61.2.195

[B37] Shapiro M. Solano D. M. Bergkamp J. J. Gebauer A. Gillian E. Lopez K. M.. (2020). Impacts of converting courses to virtual instruction midsemester at a hispanic-serving institution. J. Chem. Educ. 97, 2526–2533. 10.1021/acs.jchemed.0c00788

[B38] Sharma S. Dick G. Chin W. Land L. (2007). Self-regulation and e-learning. Eur. Conf. Inf. Syst. 2007 Proc. 45, 383–394.

[B39] Wang C. Zhao H. (2020). The impact of COVID-19 on anxiety in Chinese University students. Front. Psychol. 11:1168. 10.3389/fpsyg.2020.01168 32574244 PMC7259378

[B40] Wänke M. Hansen J. (2015). Relative processing fluency. Curr. Dir. Psychol. Sci. 24, 195–199. 10.1177/0963721414561766

