# Supplementary material for: Forced Remote Learning During the COVID-19 Pandemic in Germany: A Mixed-Methods Study on Students' Positive and Negative Expectations
Source: Front Psychol. 2021 Aug 31;12:642616. doi: 10.3389/fpsyg.2021.642616 (PMC8439379; doi:10.3389/fpsyg.2021.642616)
Supplement: Supplementary file 1 [file Data_Sheet_1.PDF]

Supplementary Material

Supplementary Table A

Distribution of statements reflecting negative effects expected from the abrupt shift from offline to online teaching and learning.

Category	Negative effect 1	Negative effect 2	Negative effect 3	Negative effect 4	Negative effect 5
Decreased quality of teaching and learning, including performance assessment	60	74	79	52	39
General decrease or lack of social interaction and communication	90	64	63	47	36
Fear of less contact, interaction with, and support from other students	58	60	48	24	18
Negative effects on course of studies, including study time extension	77	44	29	28	14
Closure of university facilities and services and hence impeded access to university resources	35	33	48	36	28
Problems with self-regulation in terms of self-studying, self-organization, self-discipline, and problems with structuring everyday life	37	47	37	28	19
Perceived uncertainty due to a lack of information	32	33	26	28	22
Increased effort required for studying in terms of time and workload	49	34	20	18	8
Less contact and interaction with lecturers and university staff and reduced feedback and support	29	26	25	21	16
Decreased motivation in study activities and learning	13	22	13	9	12
Lack of an appropriate working environment	11	21	16	8	6
Technological problems on the student side, including hardware and software	9	16	15	10	7
General problems and concerns regarding technology	11	13	6	10	8
Negative impacts of working environment on health	5	5	5	6	5
Lack of clear separation between study activities and private life	7	4	3	5	2
Financial problems	4	2	6	4	3
General negative affect	1	3	3	1	2
Technological problems regarding the stability of digital services	1	3	2	1	2
Other unspecific or rare statements not fitting any of the categories above	31	41	35	24	24
Overall	560	545	479	360	271

Note. Students were asked to report up to five negative effects (i.e., fewer than five answers were also permitted), resulting in a decreasing number of statements across effects.

Supplementary Table B

Distribution of statements reflecting positive effects expected from the abrupt shift from offline to online teaching and learning.

Category	Positive effect 1	Positive effect 2	Positive effect 3	Positive effect 4	Positive effect 5
Saving of time	172	77	40	23	13
Increased flexibility in time and work management	132	84	59	22	9
Gain in media- and study-related competences and skills	64	62	48	25	18
Hope for progress of digitalization in universities and society	38	35	26	22	21
Increased flexibility in the (asynchronous) reception and processing of course materials	20	41	26	14	6
Hope for better work-life balance due to remote learning	22	22	25	19	15
Increased quality of digital teaching and learning	12	23	19	6	3
Increased flexibility in general	30	17	8	2	4
Increased flexibility in learning location	9	19	13	8	4
Benefits for communication and interaction	1	4	6	6	5
Financial gains and savings during the challenging pandemic situation	1	8	7	3	1
Staying healthy	2	5	5	3	3
Slowing down and more sleep	5	9	3	1	0
Protection of the environment	0	1	0	4	2
Increased motivation in study activities and learning	2	2	1	0	0
Other unspecific or rare statements not fitting any of the categories above	30	46	30	29	22
Overall	540	455	316	187	126

Note. Students were asked to report up to five positive effects (i.e., fewer than five answers were also permitted), resulting in a decreasing number of statements across effects.
